# Parathyroid Apoplexy Following Cinacalcet Treatment in Primary Hyperparathyroidism

**DOI:** 10.3389/fendo.2018.00777

**Published:** 2018-12-21

**Authors:** Giulia Di Dalmazi, Cesidio Giuliani, Giorgio Napolitano

**Affiliations:** Unit of Endocrinology, Department of Medicine and Sciences of Aging and Ce.S.I.-Me.T., University of Chieti-Pescara, Chieti, Italy

**Keywords:** parathyroid apoplexy, cinacalcet, primary hyperparathyroidism, hungry bone syndrome, hypocalcemia

## Abstract

Cinacalcet, a calcimimetic drug, is considered a safe and valid option for the treatment of hypercalcemia in patients with primary hyperparathyroidism who are unable to undergo parathyroidectomy. Hypocalcemia and gastrointestinal adverse reactions are the main side effects reported in patients treated with cinacalcet. We present here the case of an 80-years-old patient with primary hyperparathyroidism treated with cinacalcet for 17 months who developed a severe and symptomatic episode of hypocalcemia requiring hospitalization 1 month after reaching a daily dose of 180 mg. Follow-up laboratory and imaging exams showed remission of primary hyperparathyroidism and disappearance of the parathyroid adenoma, suggesting a possible association between cinacalcet therapy and parathyroid infarction resulting in normalization of the elevated serum parathyroid hormone levels and severe hypocalcemia. No known cases of iatrogenic parathyroid apoplexy have thus far been described. We report here the first case of parathyroid apoplexy associated with the administration of cinacalcet in a patient with primary hyperparathyroidism. Parathyroid apoplexy features heterogeneous clinical manifestations ranging from relatively asymptomatic to potentially life-threatening cases. The occurrence of this complication should be carefully considered in patients with primary hyperparathyroidism in therapy with cinacalcet.

## Background

Primary hyperparathyroidism (PHPT) is a common endocrine disorder characterized biochemically by hypercalcemia and elevated or inappropriately normal levels of parathyroid hormone (PTH). PHPT is even more common in the elderly, with a prevalence of up to 2% (1).

It is caused in most cases (80%) by a single parathyroid adenoma, less frequently (15–20%) by a multiple gland disorder (such as multiple adenomas or multi-gland parathyroid hyperplasia), rarely (<1%) by a parathyroid carcinoma (2). In its classical form, rarely seen today, primary hyperparathyroidism features skeletal, renal, gastrointestinal, neurological, and psychiatric manifestation (2). Most patients nowadays are asymptomatic and discovered incidentally during routine laboratory testing. These asymptomatic subjects, however, may develop with time overt symptoms. Surgical removal of the parathyroid gland(s) is currently the only cure for primary hyperparathyroidism (3) but it cannot be used in a subset of patients (4) and is often deferred in elderly patients (5). For them, a valid treatment option is the administration of cinacalcet. Cinacalcet is an allosteric modulator of the calcium-sensing receptor (CaSR) that ultimately yields reduction of PTH. This drug was approved by the European Medicines Agency and the U.S. Food and Drug Administration for the treatment of hypercalcemia in hyperparathyroid patients who are unable to undergo parathyroidectomy (6).

We herein report a case of parathyroid adenoma apoplexy following cinacalcet treatment in a patient with primary hyperparathyroidism. Although spontaneous cases of parathyroid apoplexy have been previously described in primary hyperparathyroidism (7–9), this is the first case observed in association with cinacalcet treatment.

## Case Presentation

An 80-year-old white male was referred to our endocrinology outpatient clinic in December 2015 for a fracture of the left radial head, hypercalcemia (11.9 mg/dL; normal range 8.1–10.4 mg /dL), increased PTH (681 pg/ml; normal range 15–65 pg/mL), and increased alkaline phosphatase (375 U/L; normal range 40–130 U/L). Medical history was positive for hypertension and benign prostatic hyperplasia. Physical examination, including that of the cervical region, was overall normal but a 2-year history of bone pain, muscle weakness, and nephrolithiasis was noted. Renal (CKD-EPI 87, 1 ml/min/1.73 m^2^) and hepatic functions were normal (Table 1), as well as cardiovascular function. His medications included valsartan, alfuzosin, and cholecalciferol.

**Table 1:** Main laboratory data at admission and during follow-up.

	**Calcium, mg/dl** **(NR: 8.1–10.4)**	**iPTH,** **pg/mL** **(NR:15–65)**	**25-OH Vit. D, ng/mL** **(NR: > 20)**	**Creatinine, mg/dL** **(NR: 0.5–1.1)**	**Alkaline Phosphatase, U/L** **(NR: 40–130)**
December 2015 (at admission)	11.9	681	NA	0.74	375
April 2016	11.8	512	26	NA	NA
November 2016	12.4	751	23	NA	NA
February 2017	12.7	798	NA	0.73	NA
June 2017	6.3	53.6	22	0.93	411
November 2017	9.2	40.2	*28*	0.83	NA
March 2018	9.0	61.1	26	0.91	79
July 2018	9.1	61.9	NA	0.95	NA

*iPTH, intact parathyroid hormone, chemiluminescence immunoassay*.

*NA, not available; NR, normal range*.

**Figure 1:**
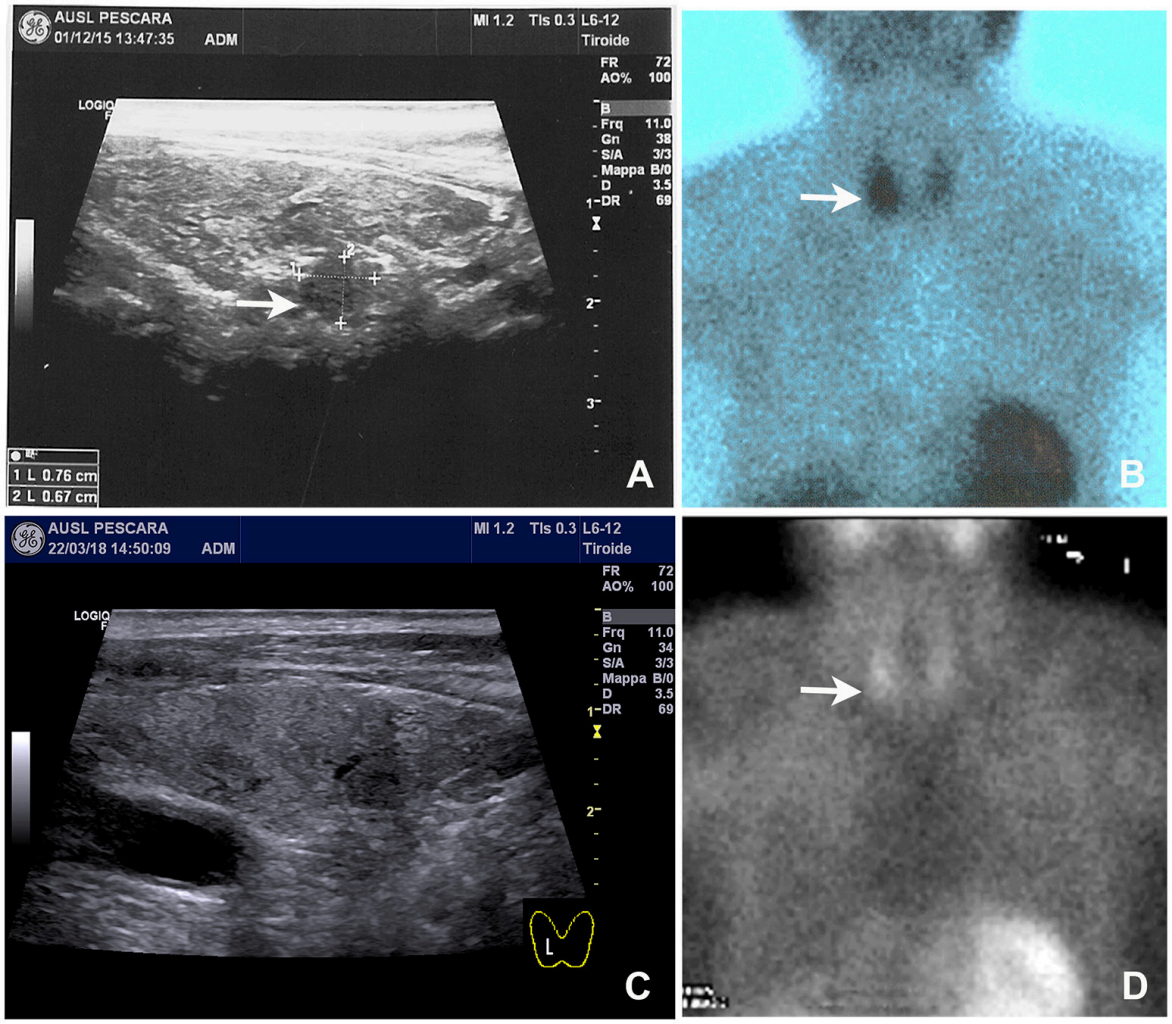
Neck ultrasound and ^99m^Tc-sestamibi scintigraphy performed on admission and 8 months after parathyroid apoplexy. **(A)** Baseline neck ultrasound showed a solid and hypoechoic 8 mm nodule located behind the right thyroid lobe (arrow). **(B)** Baseline ^99m^Tc-sestamibi scintigraphy showed an area of increased uptake (arrow). **(C)** Follow-up neck ultrasound performed 8 months later notice the near complete disappearance of the nodule. **(D)** Follow-up ^99m^Tc-sestamibi scintigraphy performed 8 months later showing a marked reduction of the uptake, although residual activity was still noticeable.

**Figure 2:**
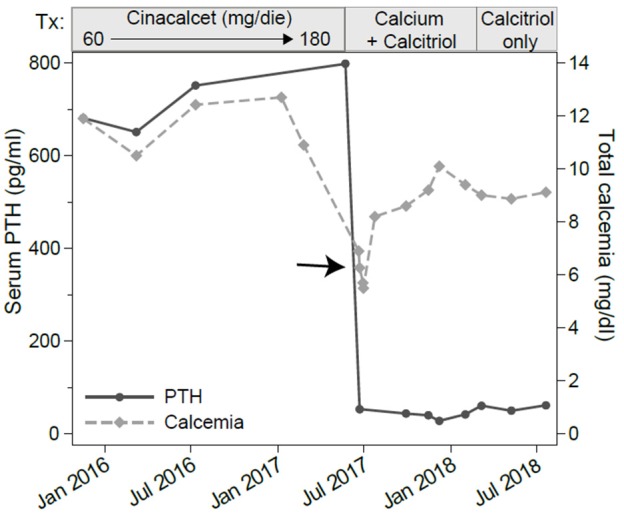
Serum levels of total calcium and parathyroid hormone (PTH). The x-axis shows the calendar time and the boxes above the graph show the treatments. Assessments were made at several time points before, during and after cinacalcet treatment. Note the marked decline in PTH and total calcium (arrow) occurring about a month after cinacalcet reached the 180 mg/dl daily dosage.

Neck ultrasound revealed a 9–mm, hypoechoic mass behind the right lobe of the thyroid gland (Figure 1A). Technetium 99m (^99m^Tc)-sestamibi scintigraphy showed a focal area of increased uptake (Figure 1B), corresponding to the ultrasound finding, thus suggesting a parathyroid adenoma. A diagnosis of primary hyperparathyroidism due to a parathyroid adenoma was, therefore, established. A parathyroidectomy was offered to the patient, but refused. In January 2016 we thus began medical treatment with cinacalcet, starting at a dose of 30 mg twice a day. This dosage was gradually increased, in the following months, to 60 mg 3 times a day as to normalize the serum calcium levels (Figure 2, left panel; Table 1).

In June 2017, 1 month after reaching a daily dose of 180 mg, the patient was admitted to the emergency room for tetany. Laboratory testing showed hypocalcemia (6.27 mg/dL), normal PTH (53.6 pg/mL), hypophosphatemia (2.7 mEq/L, normal range 3.5–5.5 mEq/L) and still increased alkaline phosphatase levels (411 U/L). Renal and hepatic functions were not impaired (Table 1). He was treated with intravenous calcium gluconate, and stopped cinacalcet treatment.

**Figure 3:**
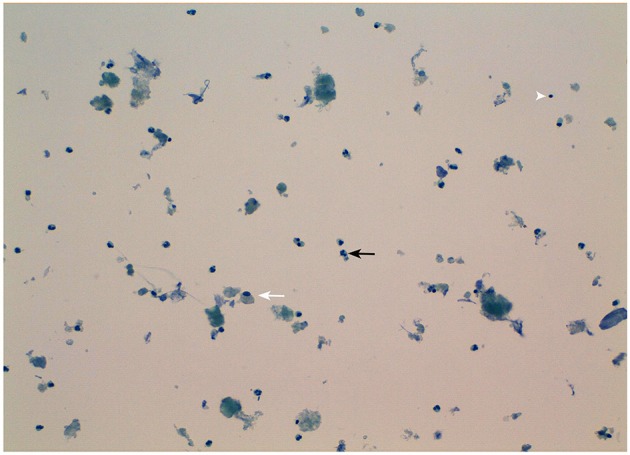
Morphological appearance of the fine-needle aspirate of the patient's parathyroid nodule. Note the presence of necrotic debris and inflammatory cells mainly consisting in neutrophils (black arrow), macrophages (white arrow), and lymphocytes (arrowhead). Original magnification 20X.

Serum calcium, surprisingly, did not rise after cinacalcet cessation, remaining around values of 6 mg/dL for about 10 days (Figure 2, arrow). Neck ultrasound confirmed the presence of a hypoechoic, irregular mass behind the thyroid right lobe, this time 17 mm in diameter. An ultrasound-guided fine needle aspiration of this lesion was performed. Cytological examination revealed basophilic amorphous material, neutrophils (Figure 3, black arrow), macrophages (Figure 3, white arrow), and lymphocytes (Figure 3, arrowhead), consistent with necrosis and inflammation. The patient was given calcium supplement (calcium carbonate, 1,000 mg twice daily) and 1,25 dihydroxyvitamin D3 (calcitriol, 0.5 μg once per day). Three months later, serum calcium normalized and PTH remained normal (44.0 pg/ml) (Figure 2). Neck ultrasound performed at the same time no further identified the parathyroid lesion (Figure 1C). Repeated ^99m^Tc-sestamibi scintigraphy showed a minute area of increased uptake, which we interpreted as a remnant adenoma, although a local inflammatory reaction could not be excluded (Figure 1D). We gradually decreased calcium treatment to a dosage of 500 mg/die and eventually stopped it in March 2018, whereas we continued calcitriol therapy (0.5 μg once per day). At the time of our latest assessment (July 2018), calcium (9.12 mg/dL), PTH (61.9 pg/mL), and alkaline phosphatase (79 U/L) were in the normal range (Table 1), clinical conditions were satisfactory except for the muscle weakness which did not improve significantly.

The above described findings suggested that the remission of primary hyperparathyroidism was caused by the apoplexy of the parathyroid adenoma.

## Discussion

Spontaneous cases of parathyroid apoplexy, were firstly described in 1946 (7) and defined pathologically by the presence of hemorrhage or infarction within the adenoma (8). Spontaneous parathyroid apoplexy occurs rarely in primary hyperparathyroidism and features heterogeneous manifestations such as cervical pain, neck mass and compression symptoms, hematoma or ecchymosis, hypercalcemic crisis due to acute PTH release, and hypocalcemia with tetany or convulsions due to auto-parathyroidectomy (8). Asymptomatic cases and potentially life-threatening manifestations are also described (9). The pathogenesis of parathyroid apoplexy is considered secondary to an imbalance between the growth of the adenoma and its blood supply. No known cases of iatrogenic parathyroid apoplexy have thus far been described. We report here the first case of parathyroid adenoma apoplexy due to a glandular infarction, as suggested by the cytological and clinical features, induced by cinacalcet therapy given for primary hyperparathyroidism.

Our patient had been given cinacalcet therapy for 17 months to control his classical symptoms of primary hyperparathyroidism. A dose of 180 mg/day was reached, since lower doses of cinacalcet failed to control the hypercalcemia (Figure 2 and Table 1). High doses of cinacalcet, although unusual, are sometimes necessary even in cases of primary hyperparathyroidism of benign etiology (10, 11). During this treatment, he developed an acute episode of severe hypocalcemia associated with drop in PTH concentration requiring hospitalization, drug discontinuation, and intravenous calcium replacement. Follow-up laboratory and imaging exams showed remission of primary hyperparathyroidism and disappearance of the parathyroid adenoma, possibly revealing an association between cinacalcet therapy and parathyroid infarction, resulting in a normalization of the elevated serum PTH levels and severe hypocalcemia. The sustained hypocalcemia, associated with the hypophosphatemia, suggested to us a mechanism akin to the “hungry bone syndrome,” which usually occurs in the context of prolonged PTH elevation followed by a marked and rapid PTH decline. Notably, the same mechanism has been described in patients with hyperparathyroidism secondary to end stage renal disease treated with cinacalcet (12).

The treatment of choice for spontaneous parathyroid apoplexy is surgery. In a few cases a conservative approach, with clinical and biochemical follow-up, is chosen. In our patient, surgery was not performed considering the favorable clinical outcome. Despite the lack of histological confirmation, an infarction of the parathyroid adenoma was strongly suggested by the acute onset of hypocalcemia, the rapid drop in serum PTH levels, and the cytological findings.

The neck ultrasound and 99mTc-sestamibi scintigraphy, further supported the occurrence of parathyroid infarction.

It is interesting to speculate on the cause of parathyroid apoplexy in our patient as it relates to the mechanism of action of cinacalcet. Cinacalcet is a calcimimetic, which, like extracellular calcium, activates the CaSR on parathyroid cells, ultimately leading to decreased synthesis and secretion of PTH. Randomized clinical trials and observational studies have shown the ability of cinacalcet to reduce PTH concentration and normalize serum calcium in patients with primary hyperparathyroidism and contraindications to surgery (13). It has been shown that cinacalcet does not improve bone mineral density or other PHPT-related symptoms, as recently reviewed by Leere et al. (13). On the contrary, bisphosphonates or denosumab protect against bone resorption (14, 15) and could have a role in the medical management of PHPT, especially in elderly patients with osteoporosis or at high risk of fracture. Cinacalcet has also shown effects on parathyroid cells proliferation. Imanishi et al. (16) demonstrated that cinacalcet suppress parathyroid cell proliferation without affecting apoptosis in a murine model of primary hyperparathyroidism. Conversely, increased parathyroid cells apoptosis has been described in glands surgically removed from secondary hyperparathyroidism patients treated with cinacalcet (17). Furthermore, *in vitro* experiments on human cultured parathyroid cells showed dose- and time-dependent increase of apoptotic cells by adding cinacalcet to the culture medium (17). Miller et al. (18) demonstrated that established parathyroid hyperplasia in uremic rats can be reversed by modulating CASR activity with cinacalcet. In addition, cinacalcet treatment in secondary hyperparathyroidism patients has been associated with a reduction in parathyroid hyperplastic volume documented by ultrasonography (19, 20).

Recently Coloma et al. (21) reported a decrease in parathyroid glandular size, evaluated by 99mTc-sestamibi, occurring in associations with the reduction of PTH levels and serum calcium concentration in a patient with asymptomatic primary hyperparathyroidism in treatment with cinacalcet.

A case of parathyroid hemorrhage after administration of cinacalcet in a patient with secondary hyperparathyroidism on hemodialysis requiring emergency surgery has been reported. Authors suggest that parathyroid hemorrhage was the consequence of a glandular degeneration caused by the administration of cinacalcet (22). Our case confirms and expands these findings by showing for the first time its occurrence in a patient with primary hyperparathyroidism.

Recurrence of primary hyperparathyroidism following initial spontaneous remission has been described (23), therefore in cases in which a conservative approach is chosen, careful clinical and biochemical follow-up is necessary.

## Conclusion

We report here the first case of parathyroid apoplexy associated with the administration of cinacalcet in a patient with primary hyperparathyroidism. The occurrence of this complication should be carefully considered in patients with primary hyperparathyroidism treated with cinacalcet. Long-term data about the management of patients with primary hyperparathyroidism treated with cinacalcet are desirable.

## Ethics Statement

This study was exempt from ethical approval procedures being a case report that describes the clinical course and outcome of a single patient who was referred to our outpatient clinic. The patient provided written consent to have his case published for the purpose to improve the medical knowledge.

## Author Contributions

GDD: clinical and endocrinological evaluation, contributions to the conception and design of the work, drafting the work, final approval of the version to be published, and agreement to be accountable for all aspects of the work in ensuring that questions related to the accuracy or integrity of any part of the work are appropriately investigated and resolved. CG: substantial contributions to the conception of the work, revising the work critically for important intellectual content, final approval of the version to be published, and agreement to be accountable for all aspects of the work in ensuring that questions related to the accuracy or integrity of any part of the work are appropriately investigated and resolved. GN: substantial contributions to the design of the work; revising the work critically for important intellectual content; final approval of the version to be published; and agreement to be accountable for all aspects of the work in ensuring that questions related to the accuracy or integrity of any part of the work are appropriately investigated and resolved.

### Conflict of Interest Statement

The authors declare that the research was conducted in the absence of any commercial or financial relationships that could be construed as a potential conflict of interest.
